# Evaluating imbalances of adverse events during biosimilar development

**DOI:** 10.1080/19420862.2016.1171431

**Published:** 2016-04-06

**Authors:** Alicia M. Vana, Amy W. Freyman, Steven D. Reich, Donghua Yin, Ruifeng Li, Scott Anderson, Ira A. Jacobs, Charles M. Zacharchuk, Reginald Ewesuedo

**Affiliations:** a Pfizer Inc, San Diego, CA, USA; b Pfizer Inc, New York, NY, USA; c Pfizer Inc., at the time of study conduct, Clinical Research and Development Consultant, Del Mar, CA, USA; d Pfizer Inc, Cambridge, MA, USA

**Keywords:** Biosimilar, breast cancer, HER2, safety, trastuzumab

## Abstract

Biosimilars are designed to be highly similar to approved or licensed (reference) biologics and are evaluated based on the totality of evidence from extensive analytical, nonclinical and clinical studies. As part of the stepwise approach recommended by regulatory agencies, the first step in the clinical evaluation of biosimilarity is to conduct a pharmacokinetics similarity study in which the potential biosimilar is compared with the reference product. In the context of biosimilar development, a pharmacokinetics similarity study is not necessarily designed for a comparative assessment of safety. Development of PF-05280014, a potential biosimilar to trastuzumab, illustrates how a numerical imbalance in an adverse event in a small pharmacokinetics study can raise questions on safety that may require additional clinical trials.

## Abbreviations

*AE*: adverse event
*AUC_inf_*: area under the serum concentration–time curve from time zero extrapolated to infinite time
*AUC_T_*: area under the serum concentration–time curve from time zero to last time point with quantifiable concentration
*CI*: confidence interval
*C_max_*: maximum serum concentration
*CTCAE*: Common Terminology Criteria for Adverse Events
*HER2*: human epidermal growth factor receptor 2
*ITT*: intent-to-treat
*mAb*: monoclonal antibody
*MedRA*: Medical Dictionary for Regulatory Activities
*mITT*: modified intent-to-treat PD, pharmacodynamics
*PK*: pharmacokinetics
*trastuzumab-EU*: trastuzumab sourced from the European Union
*trastuzumab-US*: trastuzumab sourced from the United States

## Introduction

Biologics are medicinal products produced or extracted from biological sources. Thus, biologics are distinct from pharmaceutical products that are chemically synthesized from small molecule precursors. Biologics can provide essential therapeutic options for patients with cancer or other medical conditions. Patents for several biologics have expired or will expire over the next decade, thereby allowing the development and commercialization of biologics known as “biosimilars.” It is not possible to reproduce an exact copy of a biologic; thus, biosimilars are not, and cannot be regulated as, “generic” versions of currently licensed or approved biologic (also known as “originator” or “reference”) products.

Legislation has been enacted in many countries and by the World Health Organization to provide a pathway for regulatory approval of biosimilars.^1-5^ To demonstrate biosimilarity, a sponsor must show that the proposed biosimilar product is highly similar to the reference product, with no clinically meaningful differences in safety, purity or potency of the product.^4^ Regulatory agencies recommend that sponsors of potential biosimilars use a stepwise approach to develop the data and information to support demonstration of biosimilarity. The stepwise approach involves: (1) extensive state-of-the-art structural and functional characterization studies to demonstrate similarity to the reference product; (2) nonclinical (animal) studies, including toxicity; and (3) comparative clinical studies to assess pharmacokinetics (PK)/ pharmacodynamics (PD), clinical immunogenicity, and clinical safety and effectiveness.^1,3-5^ Biosimilars are evaluated based on the totality of evidence from these studies. Here, we report how a numerical imbalance in one adverse event between arms in a PK similarity study was assessed in a separate safety study specifically designed to further characterize the observation.

## Steps in the development of a potential biosimilar

The development of PF-05280014 illustrates the biosimilar pathway. PF-05280014 is being developed as a potential biosimilar to trastuzumab, a recombinant humanized monoclonal antibody (mAb) that directly targets and selectively binds to the growth-promoting protein, human epidermal growth factor receptor 2 (HER2).^6^ Trastuzumab is licensed in the United States (Herceptin®, Genentech Inc., South San Francisco, CA, USA)^7^ and approved in the European Union (Herceptin®, Roche Registration LTD, Welwyn Garden City, UK),^8^ as well as many other countries, for the treatment of HER2-overexpressing breast and gastric cancers. In analytical studies, PF-05280014 has been shown to have an identical primary amino acid sequence as trastuzumab sourced from both the European Union (trastuzumab-EU) and the United States (trastuzumab-US).^9^ Furthermore, PF-05280014 has the same characteristics as trastuzumab-EU and trastuzumab-US with respect to in vitro binding assays and biologic functional assays.^9^ In nonclinical evaluations, PF-05280014 showed similar tumor cell growth inhibition properties and PK profiles as trastuzumab-EU and trastuzumab-US, with a low and similar incidence of anti-drug antibody development.^9^ The results of these studies supported the continued development of PF-05280014 as a proposed biosimilar for trastuzumab.

The first step in the clinical evaluation of biosimilarity is to conduct a PK similarity study in which the potential biosimilar is compared with a reference product. In such a study (REFLECTIONS B327-01; NCT01603264), PF-05280014 was compared with both trastuzumab-EU and trastuzumab-US for PK similarity (the primary objective), as well as for safety, tolerability and immunogenicity, in healthy male subjects (N = 105) (Table 1).^10^ This Phase 1, double-blind, randomized trial was limited to healthy male subjects to control variability. Evaluation of individual subject serum concentration–time data showed that the 3 products exhibited similar PK profiles, and mean serum concentration–time profiles were almost superimposable.^10^ The 90% confidence intervals (CIs) for the ratios in geometric means of maximum serum concentration (C_max_), area under the serum concentration–time curve (AUC) from time zero to the last time point with quantifiable concentration (AUC_T_), and AUC from time zero extrapolated to infinite time (AUC_inf_) were within the PK similarity acceptance criteria of 80.00%–125.00% for the comparisons of PF-05280014 to trastuzumab-EU and trastuzumab-US, and for trastuzumab-EU to trastuzumab-US.^10^ The immunogenicity and safety profiles were also comparable among the 3 drugs and consistent with previous reports for trastuzumab. All post-dose samples, except one from a subject in the trastuzumab-EU group, tested negative for anti-drug antibodies.

**Table 1.** Summary of study populations and designs.

	PK Similarity Study (REFLECTIONS B327-01)^10^	Safety Study (REFLECTIONS B327-06)
Study population	Healthy male subjects (N = 105)	Healthy male subjects (N = 162)
Study design	Phase 1, double-blind, with subjects randomized (1:1:1) to receive a single 6 mg/kg IV dose of PF-05280014, trastuzumab-US, or trastuzumab-EU	Phase 1, double-blind, with subjects randomized (1:1) to receive a single 6 mg/kg IV dose of PF-05280014 or trastuzumab-US
Primary objective	PK similarity for the comparisons of PF-05280014 to each of trastuzumab-EU and trastuzumab-US, and trastuzumab-EU to trastuzumab-US using standard 80.00% to 125.00% bioequivalence criteria	Estimate the relative risk of an abnormal body temperature compared with baseline following administration of PF-05280014 or trastuzumab-US (incidence of body temperature ≥38.0°C within 24 h after study drug administration)
Secondary objectives	Immunogenicity, assessed by measuring ADA and NAb Safety and tolerability	Additional safety evaluations

Abbreviations: ADA, antidrug antibodies; IV, intravenous; NAb, neutralizing antibodies; PK, pharmacokinetic.

Data from all 105 subjects were evaluated for safety. Adverse events (AEs) were similar across groups, and there were no serious AEs, deaths or discontinuations due to an AE. The majority of AEs were mild; the most common were infusion-related reactions (n = 30 [28.6%]), headache (n = 30 [28.6%]), chills (n = 21 [20.0%]), pyrexia (n = 15 [14.3%]) and nausea (n = 13 [12.4%]).^10^ Although AEs appeared to be evenly distributed among the 3 treatment arms in this study, a numerical imbalance was observed for pyrexia, which was experienced by 10 (28.6%), 3 (8.6%) and 2 (5.7%) subjects in the PF-05280014, trastuzumab-EU and trastuzumab-US groups, respectively.^10^ The primary objective of the study was to evaluate PK similarity of PF-05280014 to the reference products; the study was not statistically powered to evaluate similarity in safety. Therefore, it was not possible to determine if the imbalance in pyrexia was suggestive of a difference in similarity. Furthermore, it is uncertain how numerical imbalances in AEs observed during development of a biosimilar should be interpreted. Although it is expected that there will be imbalances in the number of individual AEs between the arms of a clinical study, it is unclear if such imbalances should be attributed to differences in underlying properties of the biologics being evaluated or to chance, especially in small studies that are not powered to evaluate statistically meaningful differences in AEs.

To understand if the numerical imbalance in pyrexia was real or a random occurrence, an additional study, described herein, was designed and conducted to specifically evaluate the incidence of pyrexia (using the Common Terminology Criteria for Adverse Events [CTCAE] definition for fever) (Table 1). This second study was an estimation study, the objective of which was to estimate the relative difference in pyrexia rates between the proposed biosimilar product and reference product to confirm that the large difference observed in the previous study was an aberration or a random event due to small sample size and large variability in the observed data.

## Results

A total of 162 subjects (81 per arm) were enrolled in the study and received study treatment as assigned (Table 2). Of these, 159 subjects (PF-05280014: n = 79; trastuzumab-US: n = 80) completed the study through study day 4 (primary completion) and 128 (PF-05280014: n = 63; trastuzumab-US: n = 65) completed the study follow-up through study day 50.

**Table 2.** Subject disposition.

	n (%)	
	PF-05280014	Trastuzumab-US
Assigned to study treatment		
Randomized (ITT)	81 (100.0)	81 (100.0)
Treated (mITT)	81 (100.0)	81 (100.0)
Per-protocol population	80 (98.8)	80 (98.8)
Completed through day 4 (primary completion)	79 (97.5)	80 (98.8)
Completed through day 50	63 (77.8)	65 (80.2)
Discontinued	18 (22.2)	16 (19.8)
Analyzed for safety		
Adverse events	81 (100.0)	81 (100.0)
Laboratory data	81 (100.0)	80 (98.8)

Abbreviations: ITT, intent-to-treat population; mITT, modified intent-to-treat population.

The incidence of pyrexia, with temperature ≥38.0°C as defined by CTCAE criteria, was the primary end point and AE of interest in this study. In the per-protocol population (n = 80), 5 (6.3%) and 11 (13.8%) healthy subjects in the PF-05280014 and trastuzumab-US treatment arms, respectively, experienced a body temperature ≥38.0°C. In all cases, this treatment-emergent AE was judged to be a treatment-related, infusion-related reaction and occurred within 24 h of study drug administration. Four grade 1 and one grade 2 pyrexia treatment-emergent AEs were reported in the PF-05280014 treatment arm, and 7 grade 1 and 4 grade 2 events were reported in the trastuzumab-US arm.

The relative risk of pyrexia in the per-protocol population following administration of PF-05280014 was 0.455 (90% CI: 0.198–1.057). This relative risk of pyrexia was not statistically significant. All lots of PF-05280014 and trastuzumab-US had at least one associated pyrexia AE (Table 3). Only one subject had pyrexia within the first 24 h following administration of the same lot of PF-05280014 that was used in the PK similarity study.^10^ Across treatments, the type and frequency of AEs were similar (Table 4). The severity of events was similar, and only one serious AE, which was unrelated to study drug, was reported. As expected, infusion-related reactions, which are associated with the administration of mAbs, was the most common AE. Clinical manifestations of these reactions most often consist of a symptom complex characterized by fever and chills.^11^ The safety profile was similar between the PF-05280014 and trastuzumab-US arms, with no substantial imbalance in AEs or other safety parameters observed. Because no concurrent medication was given to suppress fever, there was no difference in the use of antipyretics between groups.

**Table 3.** Number of subjects with temperature ≥38.0°C within 24 h of infusion, by lot (modified intent-to-treat population).

	PF-05280014 (n = 81)				Trastuzumab-US (n = 81)			
	Lot A (n = 27)	Lot B (n = 27)	Lot C (n = 27)	Total (n = 81)	Lot D (n = 27)	Lot E (n = 27)	Lot F (n = 27)	Total (n = 81)
n	1	3	1	5 (6.2%)	2	4	5	11 (13.6%)

Lot Numbers: Lot A = 12-003164; Lot B = 12-002983; Lot C = 12-000813; Lot D = 554763; Lot E = 554761; Lot F = 566304.

**Table 4.** Individual treatment-emergent adverse events occurring in ≥5% of subjects in either group (all-causality, mITT population).

	n (%)	
MedRA preferred term^*^	PF-05280014 (n = 81)	Trastuzumab-US (n = 81)
Infusion-related reaction	26 (32.1)	27 (33.3)
Headache	10 (12.3)	13 (16.0)^†^
Tachycardia	9 (11.1)	6 (7.4)
Chills	6 (7.4)	7 (8.6)
Pyrexia	5 (6.2)	11 (13.6)
Ocular hyperemia	3 (3.7)	5 (6.2)

* Presentation order by incidence in PF-05280014 group.

† Treatment-related adverse event incidence: n = 12 (14.8%).

Abbreviations: MedRA, Medical Dictionary for Regulatory Activities; mITT, modified intent-to-treat.

## Discussion

Designed to be “highly similar” to an approved or licensed biologic drug, biosimilars are evaluated based on extensive analytical, nonclinical and clinical data. Accordingly, some of the requirements for originator biologics, such as characterization of the target and identification of a potential mechanism of action, are not required for a biosimilar because these are presumed to be the same as those of the reference biologic. The key to the success of biosimilars is demonstration of biosimilarity with respect to the licensed or approved reference product, including head-to-head comparisons. The extensive analytical and nonclinical comparisons allow the clinical studies to be tailored and targeted so that the clinical program addresses properties that cannot be evaluated from the analytical and nonclinical comparisons, such as human PK and clinical immunogenicity, safety and effectiveness.

As part of the wider clinical development program for a potential biosimilar, the objective of a clinical PK similarity study is to evaluate the similarity between the proposed biosimilar product and the reference product using standard PK assessment criteria. Safety and immunogenicity data are also collected during a PK similarity study of a potential biosimilar; however, these data may need to be supplemented by additional clinical evaluations.^12^ In addition to being captured during a PK similarity evaluation, safety is part of the totality of evidence establishing comparability and, in general, the safety profile is expected to be similar to the reference product.^1^

In the PK similarity study of PF-05280014, a numerical imbalance was observed for the AE term “pyrexia” in the PF-05280014 arm compared with the trastuzumab-EU and trastuzumab-US arms. It was unclear if the observed imbalance was related to differences in underlying properties of the biologics being evaluated or to random chance, since this relatively small PK similarity study was not designed or powered to detect statistically meaningful differences in AEs. To address the concern that such an imbalance in pyrexia could indicate potential dissimilarities between PF-05280014 and the originator product(s), we conducted a study designed to objectively investigate this imbalance. The results from this study found that the incidence of pyrexia was numerically greater in the trastuzumab-US arm compared with the PF-05280014 arm. This numerical imbalance is the opposite of what was observed in the PK similarity study. Given the results of these 2 trials, variations in the incidence of pyrexia within 24 h of infusion appear to be due to chance in a small study population and not to any inherent difference between the drug products.

The clinical PK similarity study of PF-05280014 is not the first reported instance of a numerical imbalance in an AE reported in a study during biosimilar development. Another clinical PK similarity study compared a potential biosimilar to trastuzumab (FTMB; Synthon Biopharmaceuticals, Nijmegen, The Netherlands) vs the reference product Herceptin®.^13^ Flu-like symptoms and fatigue were reported more frequently in the healthy volunteers receiving FTMB compared with those receiving the reference product (flu-like symptoms reported in 15 [32.6%] vs 5 [10.9%] and fatigue in 11 [23.9%] vs 4 [8.7%], respectively). Nonetheless, the incidence was within the expected range when compared with patients receiving Herceptin®.

Physiochemical and biological characterization of CT-P13 (Remsima®; Celltrion, Inc., Incheon, Republic of Korea), a biosimilar infliximab, confirmed comparability between CT-P13 and the reference product.^14^ Clinical trial outcome demonstrated that, relative to the reference product, CT-P13 is highly similar in terms of PK and has comparable efficacy and safety profiles.^15,16^ In patients with rheumatoid arthritis, however, the incidence of serious AEs was greater in patients receiving CT-P13 than in those receiving the reference product (30 [10%] vs 21 [7.0%]).^16^ The imbalance was attributed to higher incidences of tuberculosis and pneumonia in the CT-P13 group, but the tuberculosis rate in patients treated with CT-P13 was comparable to those reported in historic rheumatoid arthritis studies with the originator product. Further, there was no plausible explanation for a difference in host defense from a mechanistic point of view. Based on a review of all evidence, the European Medicines Agency described the observed difference as most likely a chance finding.^17,18^

In summary, approval of a biosimilar is based on the overall assessment of biosimilarity to the reference product through robust analytical, nonclinical and clinical data. Results from our study indicate that the numerical AE imbalance in the PK similarity study appears to be due to chance and not to any inherent difference between PF-05280014 and trastuzumab-US. These data plus those from analytical, nonclinical and PK similarity studies supported proceeding with the continued development of PF-05280014 as a potential biosimilar trastuzumab. An ongoing, randomized, double-blind comparative clinical trial (NCT01989676; REFLECTIONS B327-02) is evaluating PF-05280014 plus paclitaxel vs trastuzumab-EU plus paclitaxel for first-line treatment of patients with HER2-positive metastatic breast cancer. In addition, a second, global, randomized, double-blind comparative clinical trial (REFLECTIONS B327-04; NCT02187744) evaluating PF-05280014 plus docetaxel and carboplatin vs trastuzumab-EU plus docetaxel and carboplatin in the neoadjuvant setting for breast cancer is ongoing.

## Methods

### Study design

**Figure 1.**
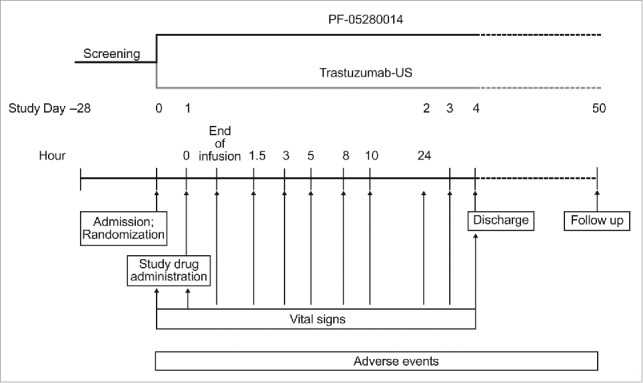
Study schema.

The design of the Phase 1, double-blind, randomized, parallel-group, single-dose clinical study is outlined in Fig. 1. The primary objective was to estimate the relative risk of an abnormal elevated body temperature compared with baseline following administration of PF-05280014 or trastuzumab-US. There were 160 subjects (80 per arm) planned for this study. Sample size was chosen to be sufficiently robust that, if an underlying difference exists, there was a high probability we would detect it, i.e., the confidence interval of the relative risk of the 2 rates would exclude one. Sample size was based on a CI precision approach, and there was no hypothesis testing as the study was designed to be an estimation study. The study is registered on ClinicalTrials.gov, identifier NCT02015156.^19^

### Subjects and treatment

The study enrolled healthy male subjects aged 18 to 55 years, with a body mass index of 17.5 kg/m^2^ to 30.5 kg/m^2^ and left ventricular ejection fraction within the normal range. This study was conducted in accordance with the Declaration of Helsinki and all local laws and regulatory requirements. All subjects provided informed consent. Subjects who had previous exposure to a biologic agent (other than an HER2 inhibitor) were eligible provided that at least 3 months had passed since the last administration of that drug. Key exclusion criteria included evidence or history of significant clinical disease or clinical finding at screening (including laboratory tests or electrocardiograms) and previous history of cancer, except for adequately treated basal cell or squamous cell carcinoma of the skin. Subjects who satisfied the eligibility criteria were admitted to the clinical research unit on day 0 prior to dosing and remained confined through the completion of procedures on day 4.

Subjects were randomized 1:1 and, following an overnight fast of at least 8 h, received a single 6-mg/kg dose of either PF-05280014 or trastuzumab-US as a 90-min intravenous infusion. To evaluate the potential influence of lot-to-lot variability, 3 lots each of PF-05280014 (lots A–C) and trastuzumab-US (lots D–F) were used in this study. PF-05280014 lot C was the same lot used in the PK similarity study.

### Concomitant medications

Subjects were to abstain from use of all concomitant medications, unless required to treat an AE. No antipyretic medications were permitted on day 0 prior to body temperature assessments, and no premedications for the purpose of suppressing infusion-related reactions were to be administered. Fever or chills were not to be routinely managed with antipyretic medications unless body temperature was ≥ 38.0°C and/or antipyretic medications were clinically indicated in the opinion of the investigator.

### Study end points and assessments

The primary end point was incidence of body temperature ≥ 38.0°C (the definition of fever in CTCAE, v4.03^20^) within 24 h after study drug administration. A series of body temperature assessments, using a consistent oral method, were obtained on day 0. To be able to characterize the potential impact of diurnal temperature variation, a minimum of 3 time points matched to the planned day 1 temperature assessments were included. On day 1, temperature assessments were performed at time 0, end of infusion and 1.5, 3, 5, 8 and 10 h after the end of infusion. On day 2, temperature was assessed 24 h after end of infusion because that is when most incidences of post-infusion pyrexia occurred after trastuzumab administration in the PK similarity study.^10^ Body temperature also was assessed once daily on days 3 and 4.

Secondary end points included incidence, severity, timing, seriousness and relationship to study therapy of the AE pyrexia (Medical Dictionary for Regulatory Activities coded term) within 24 h after study drug administration; incidence of body temperature ≥ 38.0°C and use of concomitant treatment associated with fever suppression within 24 h after study drug administration; and type, incidence, severity, timing, seriousness and relationship to study therapy of AEs, including laboratory abnormalities.

### Statistical considerations

The study was designed to estimate the relative risk in the incidence of body temperature ≥ 38.0°C within 24 h after study drug administration of PF-05280014 or trastuzumab-US to examine whether the numerical imbalance of pyrexia AEs observed in the PK similarity study was a random event. With a total sample size of approximately 160 subjects (n = 80 per treatment arm), the 2-sided 90% CI for the relative risk of incidence (PF-05280014 over trastuzumab-US) would be 0.47–2.15 if the estimated relative risk was 1.0. In this calculation, the incidence of body temperature ≥ 38.0°C was assumed to be 0.10 in the trastuzumab-US treatment group.

The intent-to-treat (ITT) population was defined as all subjects who were randomized to study treatment. The modified ITT population, defined as all subjects randomized and dosed with study treatment, was used for general safety assessments of study participants. The per-protocol population was used as the primary population for the primary end point and was defined as all subjects who were randomized, received at least 2 mg/kg (i.e., > 33%) of the planned study treatment and stayed on the study for at least 24 h after the start of infusion.
